# Enhancing COPD management in Vietnam: the strategic imperative of validating the Test of Adherence to Inhalers and the Intention of Inhaled Medication Adherence Scale

**DOI:** 10.3389/fpubh.2025.1665741

**Published:** 2025-11-03

**Authors:** Thi Quy Chu, Hoang Long Vo, Minh Sinh Do, Thi Hoa Huyen Nguyen

**Affiliations:** ^1^Nam Dinh University of Nursing, Ninh Binh, Vietnam; ^2^Tam Anh General Hospital, Hanoi, Vietnam; ^3^Department of Science, Technology, Communication and International Cooperation, E Hospital, Hanoi, Vietnam; ^4^College of Health Sciences, VinUniversity, Hanoi, Vietnam

**Keywords:** COPD, medication adherence, psychometric validation, inhaler devices, Vietnam

## Introduction

Chronic Obstructive Pulmonary Disease (COPD) represents a formidable global health challenge, exacting a heavy toll on healthcare systems and individual wellbeing worldwide (1, 2). In Vietnam, like many rapidly developing nations undergoing epidemiological transitions, the burden of COPD is escalating, fueled by a complex interplay of persistent traditional risk factors such as tobacco smoking and increasing environmental pollution (3). The cornerstone of effective COPD management—crucial for alleviating symptoms, preventing exacerbations, and improving long-term prognosis—unequivocally lies in the consistent and technically proficient use of inhaled medications (4, 5). However, suboptimal inhaler adherence remains a pervasive and deeply entrenched issue, inevitably leading to poorer clinical outcomes, increased rates of hospitalization, and a significant erosion of patients' quality of life (6). Crucially, the absence of culturally and linguistically validated measurement tools in Vietnam has historically hampered accurate assessment and effective intervention, often rendering current efforts to improve adherence inefficient. Accurately assessing, understanding, and subsequently influencing adherence behaviors are therefore paramount for both robust scientific inquiry and impactful clinical interventions. This viewpoint article critically examines the strategic importance of developing and rigorously validating culturally and linguistically appropriate inhaler adherence measurement tools, specifically the Test of Adherence to Inhalers (TAI) (7) and the Intention of Inhaled Medication Adherence Scale (IMAS) (8), within the unique Vietnamese healthcare and socio-cultural landscape. We contend that these meticulously validated instruments—a process our team aims to complete in the near future through a dedicated research endeavor—are not merely academic pursuits but represent indispensable enablers for bridging critical measurement gaps, fostering a nuanced, context-specific understanding of patient behavioral determinants, and ultimately driving substantial, measurable improvements in COPD management outcomes across Vietnam.

Although numerous instruments have been developed to assess medication adherence across chronic diseases, many of the most widely used scales are generic rather than therapy-specific. The Medication Adherence Report Scale (MARS) and the Adherence Starts with Knowledge (ASK-20) questionnaire, for example, have been frequently applied in chronic illness research and clinical practice. Their strengths lie in brevity, ease of administration, and broad applicability across diverse therapeutic areas. However, these tools were not originally designed for inhaler-based therapies and therefore may not capture critical dimensions such as inhaler technique, device-specific barriers, or the distinction between intentional and unintentional non-adherence. This limits their ability to provide a nuanced understanding of adherence in COPD populations, where incorrect technique and motivational barriers often coexist and jointly undermine treatment outcomes (9, 10).

By contrast, the Test of Adherence to Inhalers (TAI) and the Intention of Inhaled Medication Adherence Scale (IMAS) were specifically developed to address these limitations. The TAI, validated across multiple international COPD and asthma cohorts, directly evaluates inhaler-related behaviors and distinguishes between active (deliberate) and passive (unintentional) forms of non-adherence, thereby offering practical clinical insights into patients' real-world use of inhalers (7, 11). Complementing this, the IMAS is theoretically anchored in the Theory of Planned Behavior (TPB) and systematically captures the cognitive, attitudinal, and social determinants that shape patients' intentions to adhere. By exploring constructs such as attitudes toward treatment, perceived social norms, and perceived behavioral control, the IMAS sheds light on the psychological and motivational drivers of adherence (8, 12).

Taken together, TAI and IMAS offer a uniquely comprehensive framework: the former captures what patients do with their inhalers, while the latter provides insight into why they do it. This combination is particularly relevant in the Vietnamese context, where cultural, social, and health literacy factors strongly influence patient behaviors. The integration of these two instruments, once rigorously validated, therefore holds strong potential to guide both evidence-based research and the design of culturally tailored interventions for COPD management in Vietnam.

## The unmet need for culturally congruent adherence measurement in Vietnam

Recent epidemiological and cost studies indicate that COPD imposes a substantial clinical and economic burden in Vietnam. Population-based surveys estimate COPD prevalence in Vietnamese adults at approximately 7%−10%, with variation by region and sex (13–15). Acute exacerbations are common and are the principal driver of direct medical costs. In a tertiary-hospital sample of patients admitted with AECOPD in Hanoi, the mean total hospitalization cost per episode was 18.3 million VND (≈USD 780–800 in 2018), with medication accounting for the largest share of inpatient expenses (16). In outpatient settings, a multicenter study across three clinics in northern Vietnam found that 52.9% of patients experienced at least one exacerbation in the prior year and 75.7% of those exacerbations required hospitalization; mean annual costs varied by care pathway from roughly USD 306 for routine outpatients to USD 1,275 for standard hospital admission and USD 2,132 for emergency-department cohorts (17). These Vietnamese findings are consistent with regional and global evidence showing that hospital admissions and exacerbation management are the single largest contributors to COPD costs. A recent systematic review likewise underscores hospitalization as the dominant cost component and documents wide variation in per-patient costs across settings (18). Taken together, these data show a pattern familiar across the Asia–Pacific region: high prevalence, frequent exacerbations, and disproportionate inpatient costs that translate into substantial out-of-pocket spending and productivity losses for patients and caregivers. In this context, suboptimal adherence and incorrect inhaler technique, both documented problems in Vietnam, are not merely clinical issues but key economic drivers: poor adherence increases exacerbation risk, hospital use, and therefore costs.

Despite the critical role of inhaler adherence in COPD management, a significant unmet need persists in Vietnam for culturally congruent and validated measurement tools. This imperative stems directly from the nation's distinctive demographic, socio-economic, and healthcare realities. Vietnam is currently undergoing a pronounced demographic shift, characterized by a rapidly aging population. This demographic segment is disproportionately affected by COPD, a chronic, progressive disease linked to both age-related physiological decline and cumulative exposure to pulmonary irritants. Concurrently, the nation continues to grapple with historically high rates of male smoking, alongside escalating concerns regarding ambient air pollution and occupational exposures, all contributing significantly to the escalating burden of respiratory ailments (3). Within this broad patient population, pronounced heterogeneity exists concerning educational attainment, socioeconomic status, and health literacy levels (19, 20). These multifaceted factors profoundly influence individuals' capacity to comprehend complex medical information, master the intricate techniques required for correct inhaler use, and shape their fundamental beliefs about medication efficacy and necessity.

In the absence of culturally and linguistically appropriate adaptation, generic, internationally developed adherence scales often prove inadequate or even misleading. They may contain idiomatic expressions that defy direct translation, conceptual frameworks that lack cultural resonance, or implicit assumptions about healthcare access, patient autonomy, or illness perceptions that simply do not align with the Vietnamese context. For instance, a question assuming direct patient questioning of a physician's advice might misrepresent adherence in a culture where deference to authority is common. Such intrinsic discrepancies can inevitably lead to misinterpretation by respondents, biased or unreliable data collection, and, consequently, profoundly inaccurate assessments of actual adherence. Beyond mere linguistic translation, the rigorous validation of scales like TAI and IMAS demands a profound, context-specific understanding of local nuances. This process ensures that the instruments genuinely capture real-world behavioral patterns and their underlying determinants in a manner that is both comprehensible to the patient and conceptually relevant to the local healthcare context. Only then can these tools form a reliable foundational pillar for evidence-based clinical practice, targeted public health interventions, and meaningful research in Vietnam. Without such tailored tools, efforts to improve adherence are inherently vulnerable to being misdirected, inefficient, or even counterproductive, thereby impeding meaningful progress in national COPD care strategies.

## Methodological rigor and conceptual robustness of TAI and IMAS in the Vietnamese context

The proposed meticulous validation of TAI and IMAS in Vietnam is poised to unequivocally confirm their conceptual soundness and methodological rigor, significantly enhancing their practical utility within the local healthcare paradigm. The TAI, internationally recognized for its direct assessment of practical adherence behaviors (7, 11), is expected to demonstrate a remarkably clear and consistent factor structure even after extensive and sensitive cultural adaptation for the Vietnamese population. This robust internal structure typically distinguishes between active non-adherence, driven by conscious patient decisions or deeply held perceptions (e.g., intentional skipping due to concerns about side effects, perceived financial barriers, or a subjective assessment of medication necessity), and passive non-adherence, which more commonly stems from unintentional factors like simple forgetfulness, busy schedules, or subtle errors in inhaler technique. Such precise structural integrity is paramount, as it will enable clinicians and researchers to accurately identify the specific dimensions of non-adherence, thereby facilitating the development of highly targeted and individualized intervention strategies.

**Figure 1:**
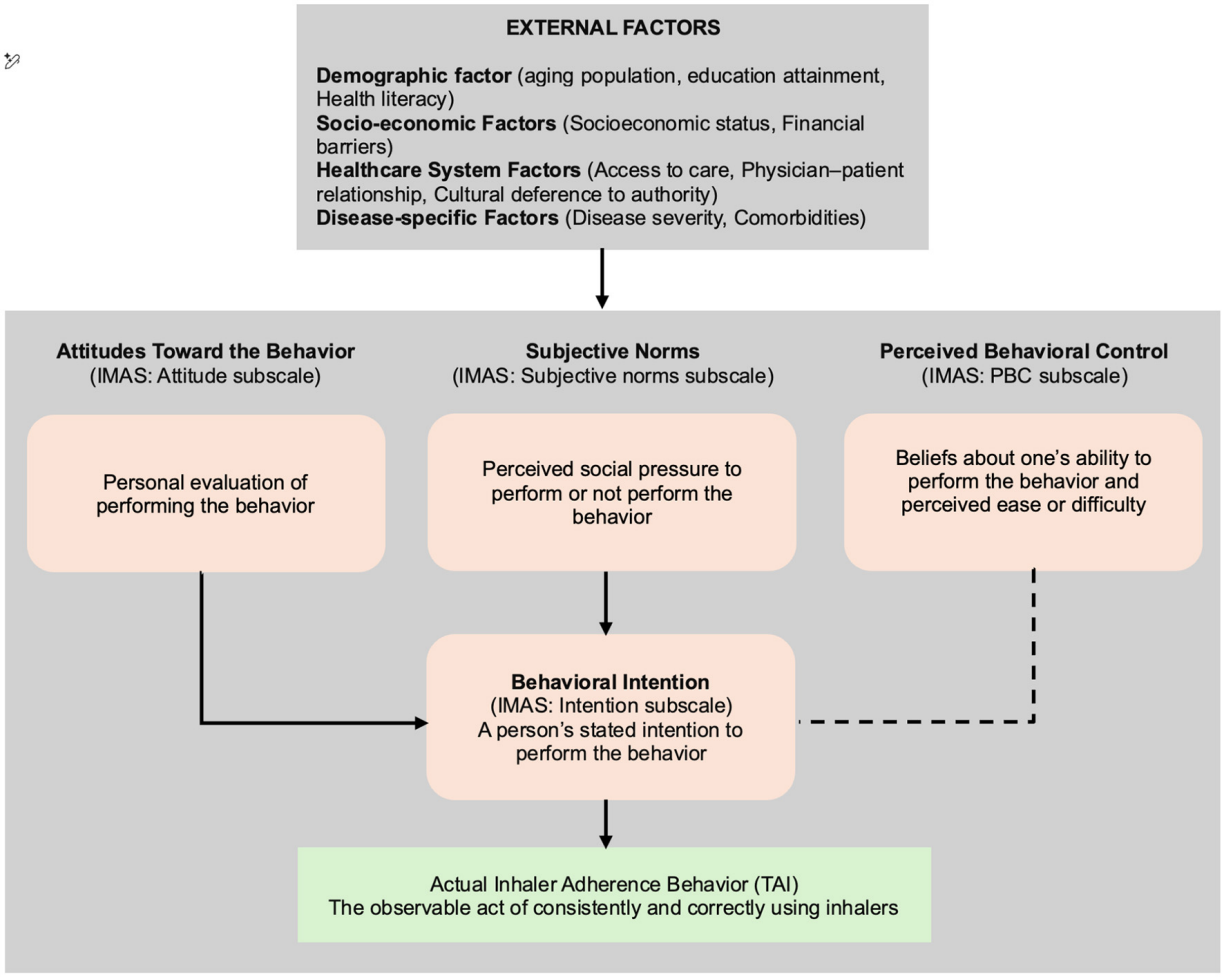
Conceptual framework of the Intention of Inhalaled Medication Adherence Scale based on the Theory of Planned Behavior.

Concurrently, the IMAS, theoretically anchored in Ajzen's influential Theory of Planned Behavior (TPB) (8, 12), is also anticipated to exhibit compelling construct validity within the Vietnamese context. The TPB posits that an individual's behavioral intention, serving as a proximal predictor of actual behavior, is collectively influenced by three fundamental determinants: their attitude toward the behavior (personal evaluation), subjective norms (perceived social pressure), and perceived behavioral control (belief in one's ability to perform the behavior). The validation of IMAS is expected to robustly confirm that its constituent components—meticulously designed to reflect these precise TPB constructs—align seamlessly with the theoretical framework, thereby affirming its profound capacity to delve into the intricate cognitive and motivational antecedents of adherence behavior among Vietnamese patients. This insight will be crucial for designing culturally tailored educational and behavioral interventions that resonate with patients' personal beliefs and social contexts. This remarkable consistency with the original psychological model, even after rigorous cross-cultural translation and adaptation, strongly underscores the universality of these core psychological constructs in explaining complex health behaviors. The synergistic application of TAI (directly measuring overt adherence behaviors) and IMAS (meticulously exploring underlying cognitive, affective, and volitional drivers) thus offers a holistic, multifaceted, and deeply insightful approach to understanding the complex nature of inhaler adherence. This combined perspective will be invaluable, empowering healthcare professionals and researchers to not only identify *what* patients are doing with their inhalers but, more importantly, to discern *why* these behaviors occur, thereby paving a clear pathway for the design and implementation of highly personalized, empathetic, and ultimately more effective interventions. This relationship is summarized in the conceptual framework of the Intention of Inhaled Medication Adherence Scale based on the Theory of Planned Behavior (Figure 1).

## Establishing robust reliability for sustainable clinical and research application

For any psychometric assessment tool, robust reliability is paramount for ensuring both scientific credibility and practical utility, encompassing internal consistency and temporal stability (21). For the Vietnamese versions of TAI and IMAS, the upcoming meticulous and exhaustive validation process is expected to unequivocally confirm their high levels of reliability, thereby firmly establishing them as exceptionally trustworthy instruments for consistent and dependable application across diverse clinical and research settings throughout Vietnam. High internal consistency, typically quantified using statistical measures like Cronbach's Alpha, is anticipated to provide compelling assurance that all individual items within each scale coherently measure the same underlying construct (22). This inherent methodological soundness will guarantee that the assessment of inhaler adherence is uniform, coherent, and highly dependable across the various questions comprising the same scale, minimizing measurement error.

Beyond this internal cohesion, the anticipated demonstration of test-retest reliability—which critically assesses the stability of the measures when administered repeatedly over a predefined time interval—will be paramount for practical application (23). This attribute will confirm that the scales yield consistent results when re-administered to the same individuals, assuming no substantive change in their actual adherence behavior. Such temporal stability is particularly vital for the design and execution of longitudinal studies, for the robust evaluation of intervention efficacy over time, and for routine, reliable monitoring of patient progress in ongoing clinical practice. A particularly striking and encouraging finding from previous comprehensive validation processes for these instruments is the remarkable maintenance of strong reliability even when administered to particularly vulnerable patient sub-populations, notably older adults or those with lower educational attainment (24, 25). These demographic groups are frequently challenging to assess accurately due to potential cognitive difficulties, varying levels of health literacy, or socio-cultural factors that may influence self-reporting. The expected robustness of TAI and IMAS within these diverse sub-populations will stand as a powerful testament to the meticulousness of the linguistic and cultural adaptation process, alongside the inherent clarity and simplicity of the translated language. This clarity effectively transcends potential educational and age-related barriers, thereby maximizing respondent comprehension and engagement. This robust reliability profile, which is either comparable to or, in certain aspects, even surpasses international benchmarks (26–28), will significantly bolster confidence in utilizing TAI and IMAS for precise patient assessment, facilitating informed clinical decision-making, enabling rigorous evidence generation, and ultimately fostering a more systematic approach to patient care within Vietnamese healthcare settings.

## Paving the way forward: unaddressed questions and research imperatives

While the anticipated successful and rigorous validation of TAI and IMAS in Vietnamese will undoubtedly represent a pivotal and commendable milestone in advancing COPD care within the country, it is crucial to acknowledge that this initial achievement also illuminates several critical avenues warranting further strategic exploration. Fully harnessing the transformative potential of these instruments in optimizing patient outcomes necessitates a sustained and multifaceted research agenda.

Firstly, recognizing that the initial validation efforts will likely predominantly rely on cross-sectional study designs—which are undeniably necessary for establishing foundational psychometric properties such as reliability and initial validity—future research must unequivocally prioritize longitudinal studies. This temporal dimension is paramount because adherence behaviors are not static; they fluctuate over time due to various patient-specific, environmental, and treatment-related factors. Longitudinal investigations will rigorously evaluate the scales' sensitivity to capture these dynamic changes over extended periods, providing insights into patterns of decline or improvement in adherence. This perspective is indispensable not only for tracking the natural evolution of adherence patterns but also for identifying critical inflection points where adherence might falter, allowing for proactive intervention. Furthermore, it will enable a robust assessment of the real-world effectiveness of educational or behavioral interventions aimed at improving adherence, moving beyond snapshots to demonstrate sustained impact. This deeper temporal understanding is vital for developing truly adaptive, responsive, and ultimately sustainable adherence support programs tailored to the long-term needs of COPD patients.

Secondly, although the initial sample populations for validation will provide a solid foundational understanding, expanding these efforts to encompass broader and more heterogeneous patient populations across a wider geographical distribution will significantly strengthen the generalizability and external validity of these instruments. Vietnam, with its diverse geographic and socioeconomic landscapes, presents unique challenges and opportunities. Future studies should compare outcomes in urban vs. rural settings, across different hospital tiers (central vs. provincial vs. district), or engage patients with diverse levels of disease severity, comorbidities, and socio-economic backgrounds. This comprehensive and inclusive approach will ensure that TAI and IMAS are truly applicable and representative across the vast and varied spectrum of COPD patients encountered throughout Vietnam, reflecting the nation's unique healthcare pluralism and diverse patient experiences. Such expanded validation will enhance the confidence in applying these tools universally across different clinical contexts and patient demographics within the country.

Thirdly, a crucial future imperative involves seamlessly integrating these invaluable self-reported measures with objective adherence monitoring tools. In many clinical settings globally, the absence of a universally accepted “gold standard” for adherence measurement underscores the necessity of this synergistic approach. While self-report measures are practical and cost-effective, they are susceptible to recall bias and social desirability. Incorporating objective data sources, such as electronic inhaler devices that precisely record dosage and timing, smart pillboxes, or even biochemical markers where feasible, would provide crucial supplementary and corroborative data. This multi-modal assessment strategy would not only confirm self-reported adherence but, more critically, would enable a far more rigorous evaluation of the predictive validity of TAI and IMAS. This would involve establishing how well these scales correlate with actual medication intake and, most importantly, with tangible clinical outcomes such as reduced exacerbation rates, improved lung function, enhanced quality of life, and decreased healthcare utilization. Such an integration would profoundly elevate the utility of these scales beyond mere descriptive assessment to truly impactful clinical and research applications, offering a comprehensive and nuanced picture of patient behavior and its direct health consequences.

Finally, future research should also strategically delve deeper into the identification and systematic mitigation of specific cultural, socio-economic, or systemic barriers to adherence that may be uniquely revealed or illuminated by these newly validated scales. For instance, deeply ingrained cultural beliefs surrounding chronic illness, the perceived role of traditional healing practices alongside modern medicine, specific family dynamics influencing medication regimens, or even structural limitations within the healthcare delivery system (e.g., access to pharmacies, cost of medication) might influence adherence in ways not yet fully captured by existing theoretical models. Addressing these nuanced influences through targeted qualitative, ethnographic, and mixed-methods research will be instrumental in further refining both the instruments themselves and the culturally tailored interventions designed to optimize adherence, ensuring they are not only effective but also sensitive to the Vietnamese context.

## Conclusions

The forthcoming rigorous validation of TAI and IMAS in Vietnamese will be a critical step, more than just a technical accomplishment, significantly advancing COPD care in the country. These psychometrically sound instruments will provide the essential methodological rigor required to accurately assess inhaler adherence, thereby laying a robust foundation for data-driven, evidence-based interventions. By continuing to refine, integrate, and apply these critical tools within an expanded and ambitious research framework, Vietnam is exceptionally well-positioned to make substantial and lasting strides toward optimizing patient outcomes, significantly reducing the overall disease burden, and concurrently setting a compelling international precedent for the development and deployment of culturally sensitive, scientifically rigorous healthcare measurement in rapidly evolving healthcare systems.
